# Supplementary material for: Lung Resident T Cells Across the Spectrum of TB Infection and Disease
Source: bioRxiv. 2026 Aug 14:2026.08.10.743910. Preprint. [Version 1] doi: 10.64898/2026.08.10.743910 (PMC13640633; doi:10.64898/2026.08.10.743910)
Supplement: Supplementary file 1 [file media-14.pdf]

a)

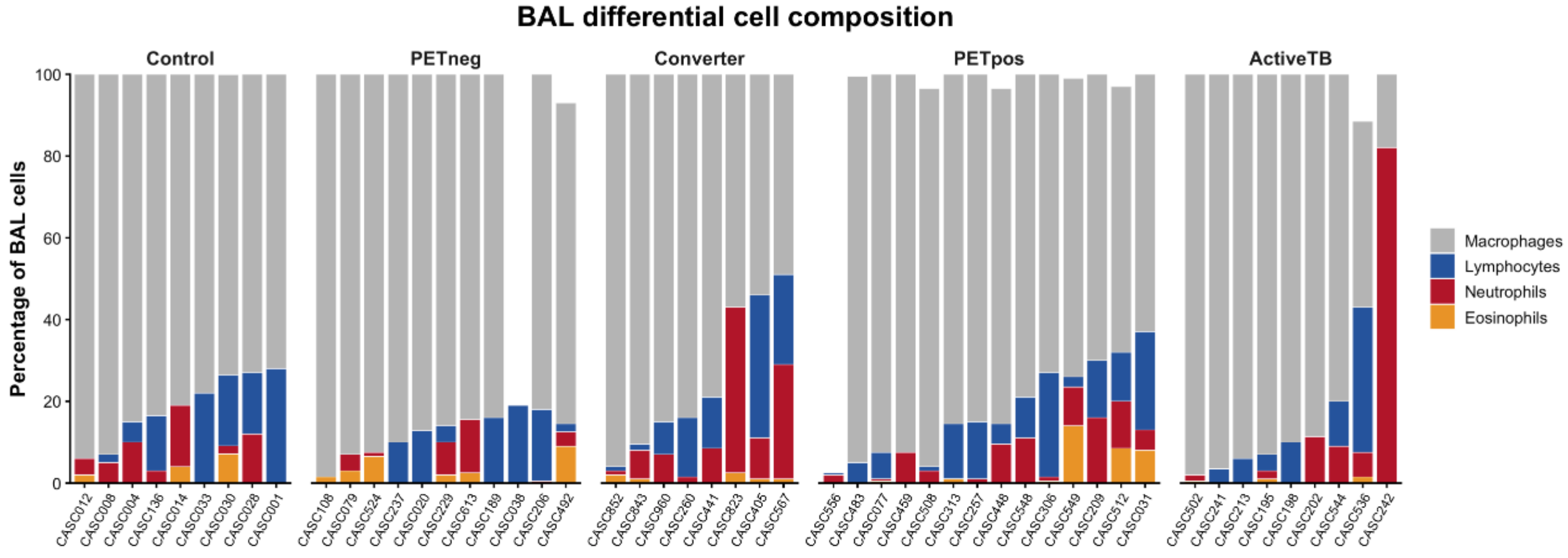
BAL differential cell composition
Control
PETneg
Converter
PETpos
ActiveTB
Percentage of BAL cells
100
80
60
40
20
0
Macrophages
Lymphocytes
Neutrophils
Eosinophils
CASC012
CASC008
CASC004
CASC136
CASC014
CASC033
CASC030
CASC028
CASC001
CASC108
CASC079
CASC524
CASC237
CASC020
CASC229
CASC613
CASC189
CASC038
CASC206
CASC492
CASC852
CASC843
CASC960
CASC260
CASC441
CASC823
CASC405
CASC567
CASC556
CASC483
CASC077
CASC459
CASC508
CASC313
CASC257
CASC448
CASC548
CASC306
CASC549
CASC209
CASC512
CASC031
CASC502
CASC241
CASC213
CASC195
CASC198
CASC202
CASC544
CASC536
CASC242

b)

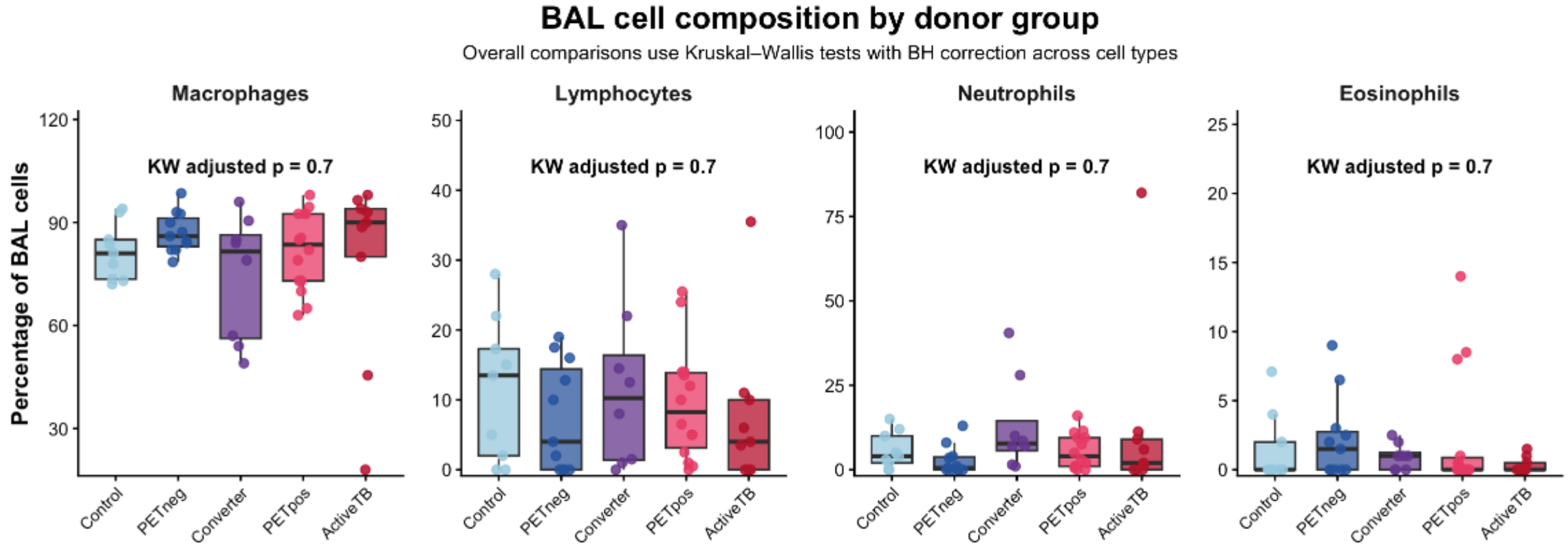
BAL cell composition by donor group
Overall comparisons use Kruskal–Wallis tests with BH correction across cell types
Macrophages
Lymphocytes
Neutrophils
Eosinophils
KW adjusted p = 0.7
Percentage of BAL cells
Control
PETneg
Converter
PETpos
ActiveTB

c)

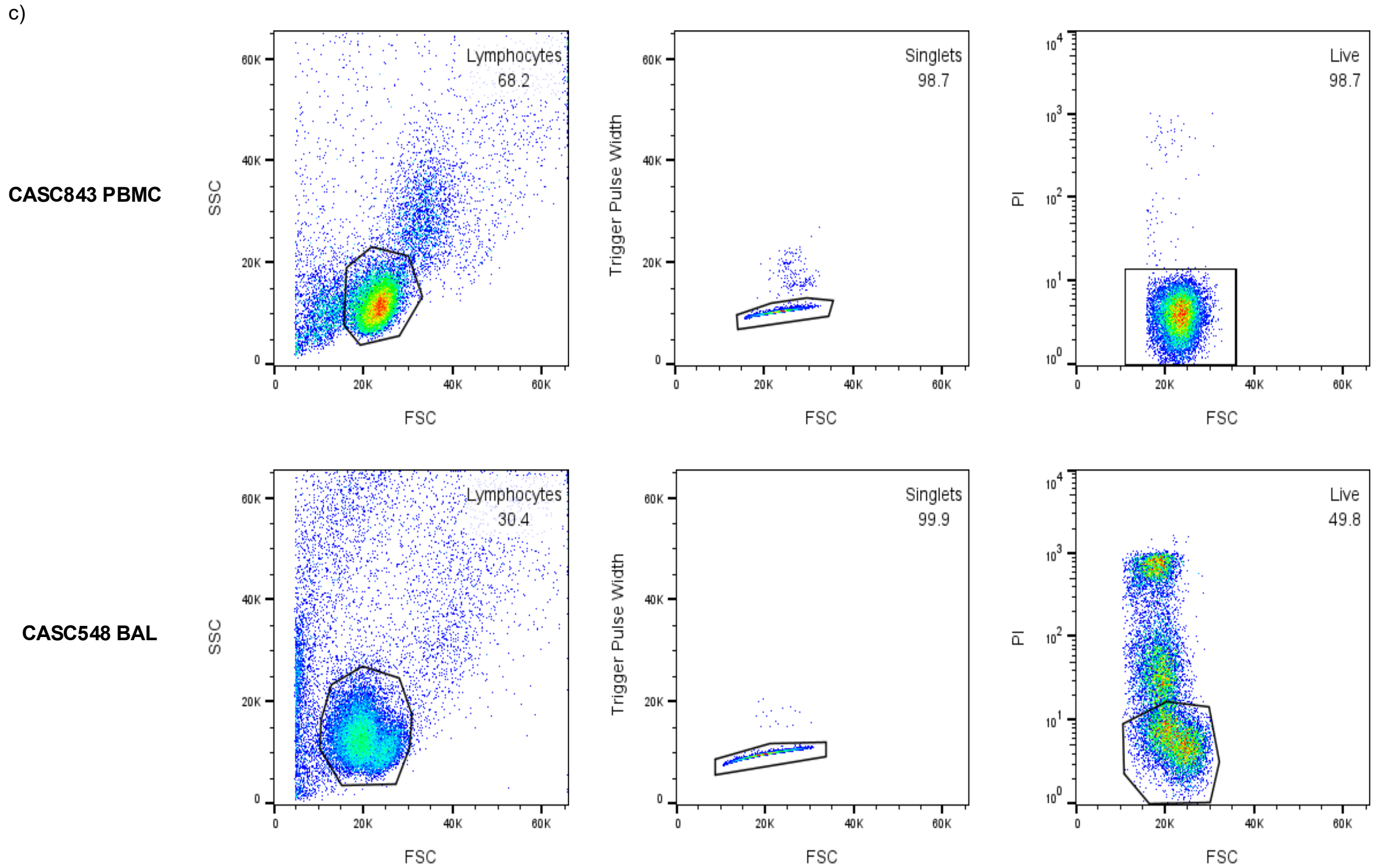
CASC843 PBMC
CASC548 BAL
SSC
FSC
Trigger Pulse Width
PI
Lymphocytes
68.2
Singlets
98.7
Live
98.7
Lymphocytes
30.4
Singlets
99.9
Live
49.8

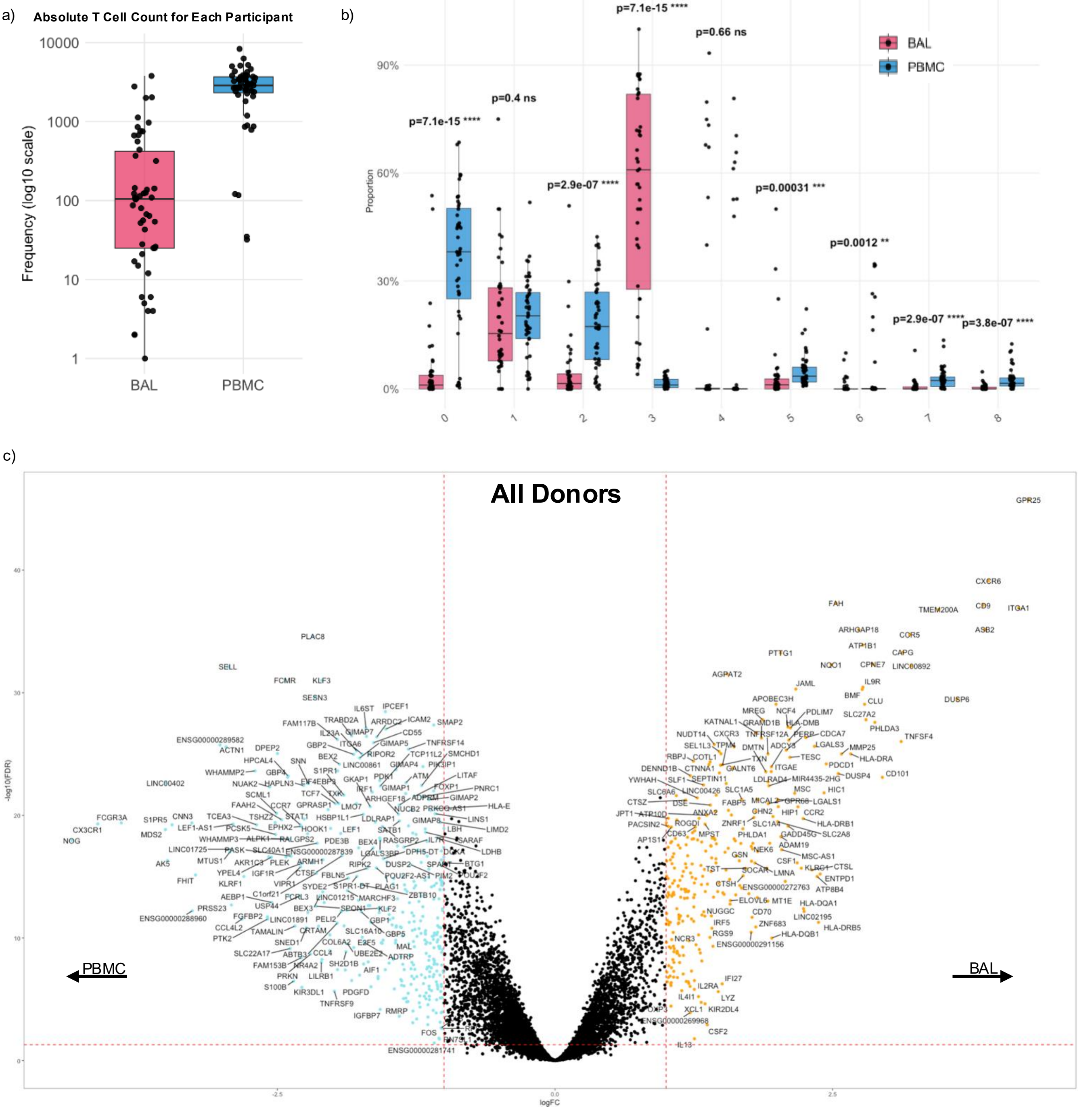
a)
Absolute T Cell Count for Each Participant
Frequency (log10 scale)
10000
1000
100
10
1
BAL
PBMC
b)
Proportion
90%
60%
30%
0%
p=7.1e-15 ****
p=0.4 ns
p=2.9e-07 ****
p=7.1e-15 ****
p=0.66 ns
p=0.00031 ***
p=0.0012 **
p=2.9e-07 ****
p=3.8e-07 ****
0
1
2
3
4
5
6
7
8
BAL
PBMC
c)
All Donors
-log10(FDR)
logFC
PBMC
BAL

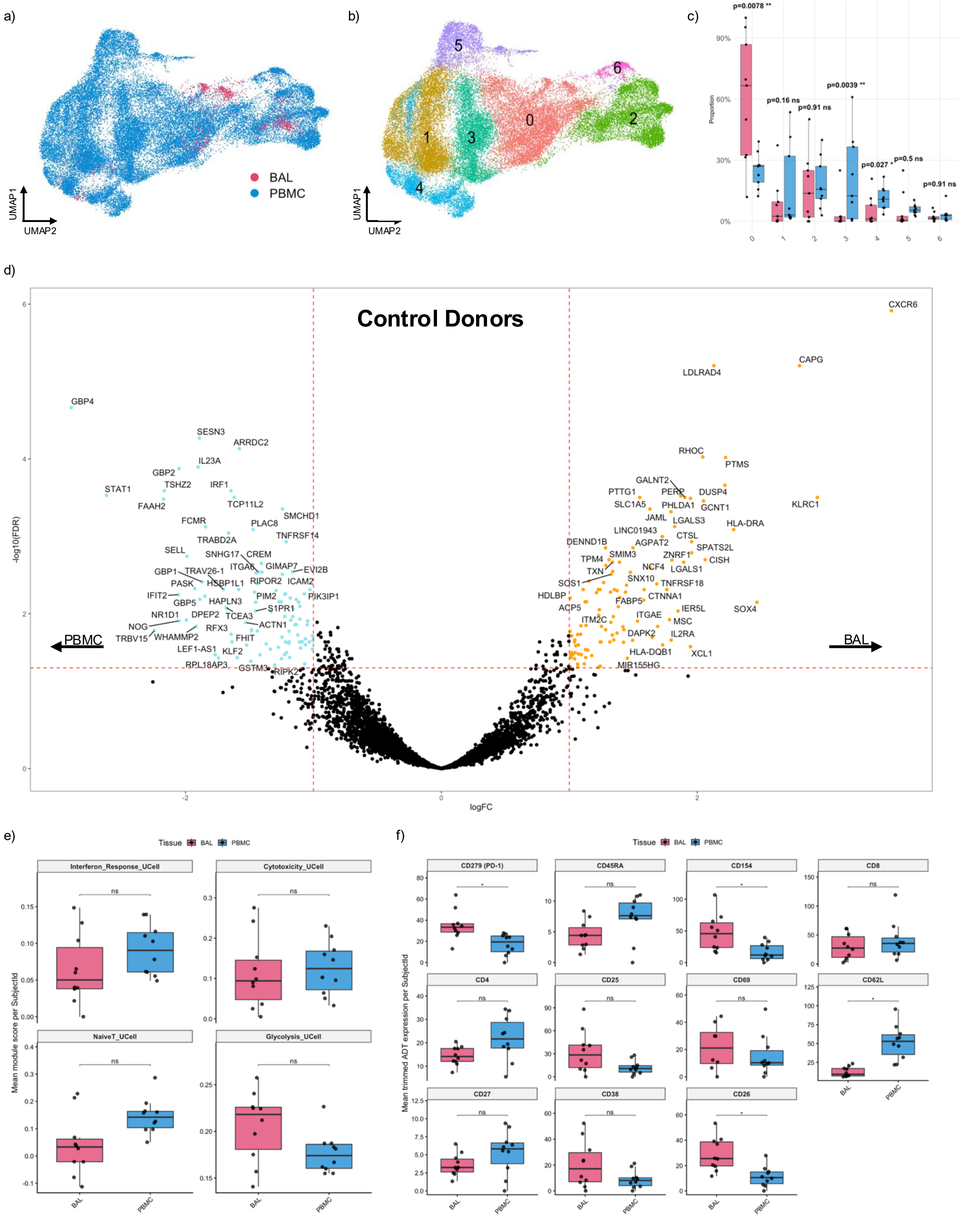

a)
BAL
PBMC
UMAP1
UMAP2
b)
5
6
0
1
3
2
4
UMAP1
UMAP2
c)
p=0.0078 **
p=0.16 ns
p=0.91 ns
p=0.0039 **
p=0.027 *
p=0.5 ns
p=0.91 ns
Proportion
90%
60%
30%
0%
0
1
2
3
4
5
6
d)
Control Donors
CXCR6
CAPG
LDLRAD4
GBP4
SESN3
ARRDC2
IL23A
GBP2
TSHZ2
IRF1
STAT1
FAAH2
TCP11L2
SMCHD1
FCMR
PLAC8
TNFRSF14
TRABD2A
SELL
SNHG17
CREM
GBP1
TRAV26-1
ITGA6
GIMAP7
EVI2B
PASK
HSBP1L1
RIPOR2
ICAM2
IFIT2
PIM2
PIK3IP1
GBP5
HAPLN3
S1PR1
NR1D1
DPEP2
TCEA3
NOG
RFX3
ACTN1
TRBV15
WHAMMP2
FHIT
LEF1-AS1
KLF2
RPL18AP3
GSTM3
RIPK2
RHOC
PTMS
GALNT2
PTTG1
PERP
DUSP4
SLC1A5
PHLDA1
GCNT1
KLRC1
JAML
LGALS3
HLA-DRA
LINC01943
CTSL
DENND1B
AGPAT2
SPATS2L
TPM4
SMIM3
ZNRF1
CISH
TXN
NCF4
LGALS1
SOS1
SNX10
TNFRSF18
HDLBP
CTNNA1
ACP5
FABP5
IER5L
SOX4
ITM2C
ITGAE
MSC
DAPK2
IL2RA
HLA-DQB1
XCL1
MIR155HG
PBMC
BAL
-log10(FDR)
logFC
-2
0
2
e)
Tissue BAL PBMC
Interferon_Response_UCell
Cytotoxicity_UCell
NaiveT_UCell
Glycolysis_UCell
ns
Mean module score per SubjectId
BAL
PBMC
f)
Tissue BAL PBMC
CD279 (PD-1)
CD45RA
CD154
CD8
CD4
CD25
CD69
CD62L
CD27
CD38
CD26
*
ns
Mean trimmed ADT expression per SubjectId
BAL
PBMC

a)

Cluster	Cell Type
0	Predominantly CD4
1	Predominantly CD8
2	Mixed
3	Double Negative
4	Predominantly CD4
5	Predominantly CD4
6	Double Negative
7	Predominantly CD8
8	Predominantly CD4

b)

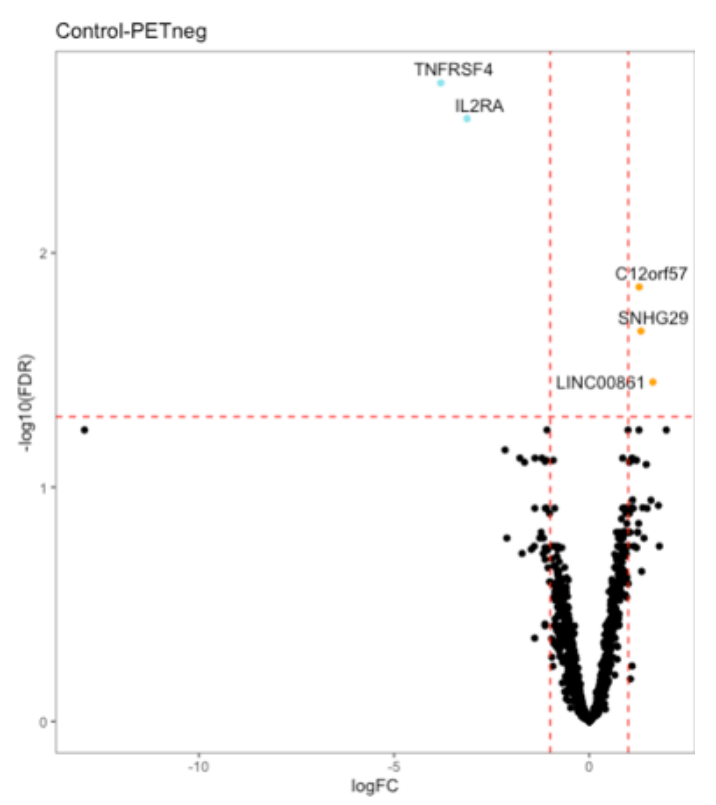

c)

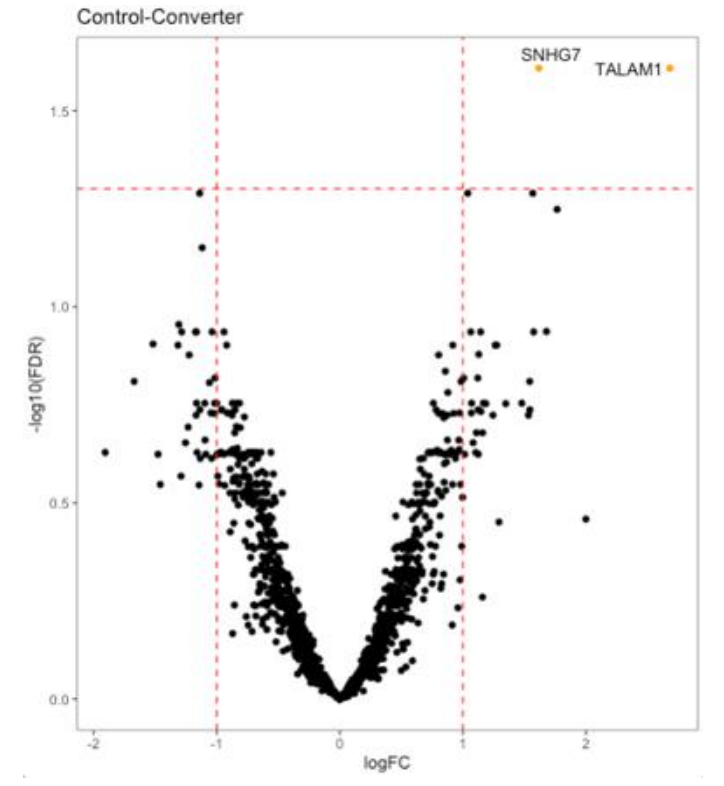

d)

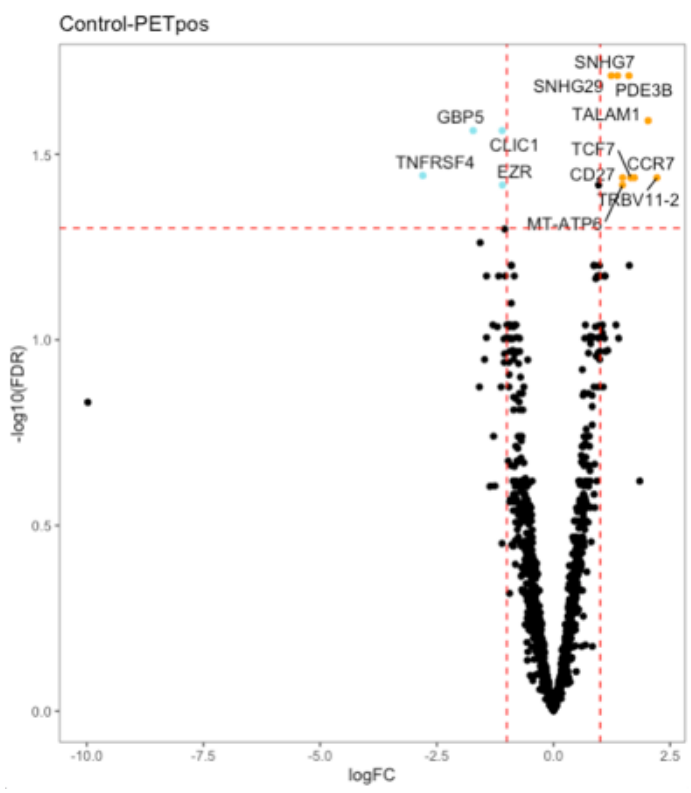

e)

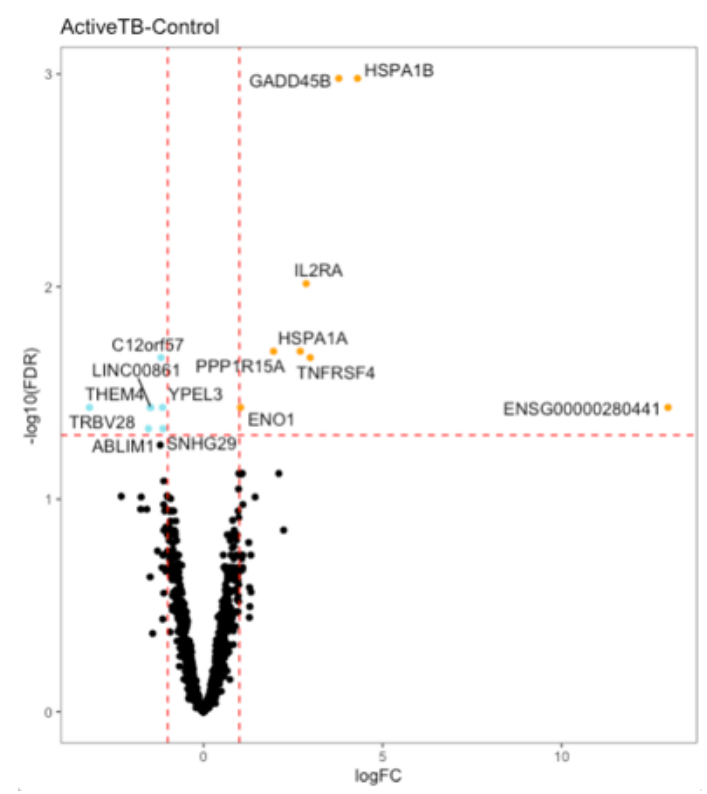

f)

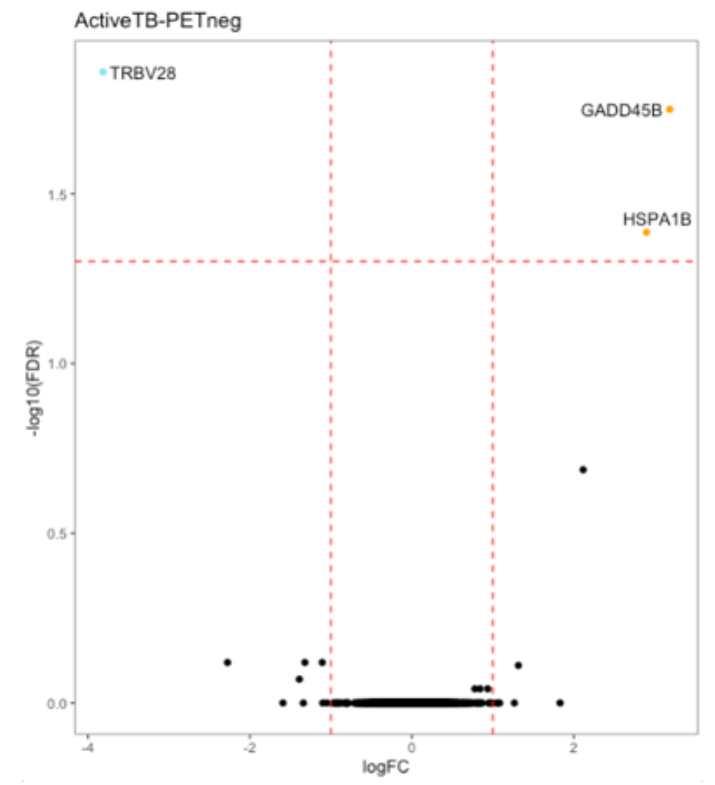

g)

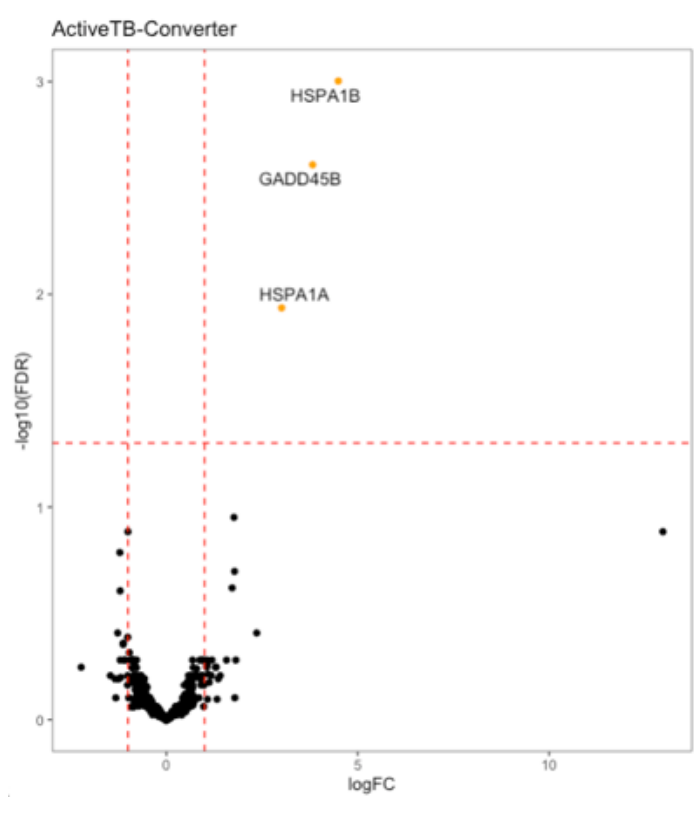

h)

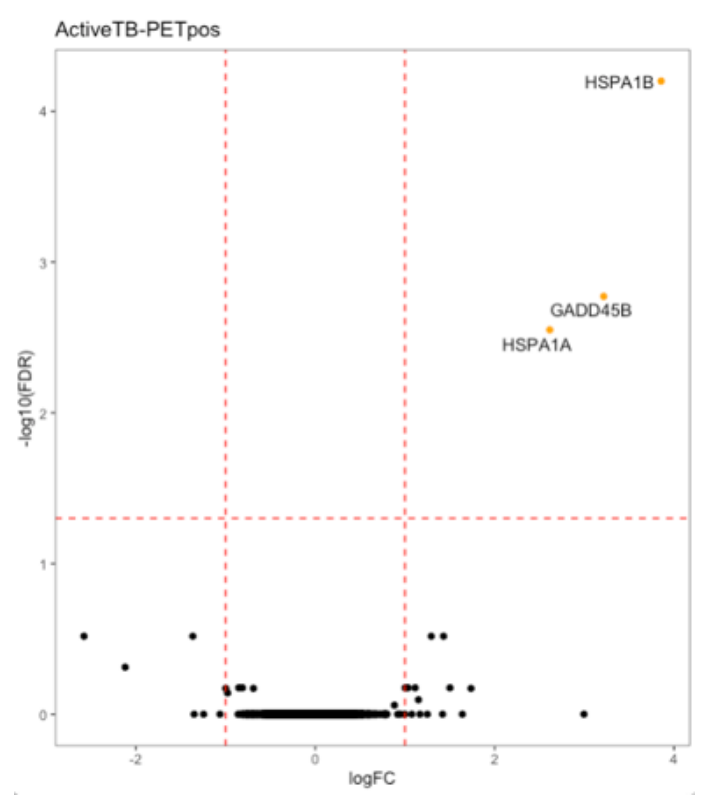

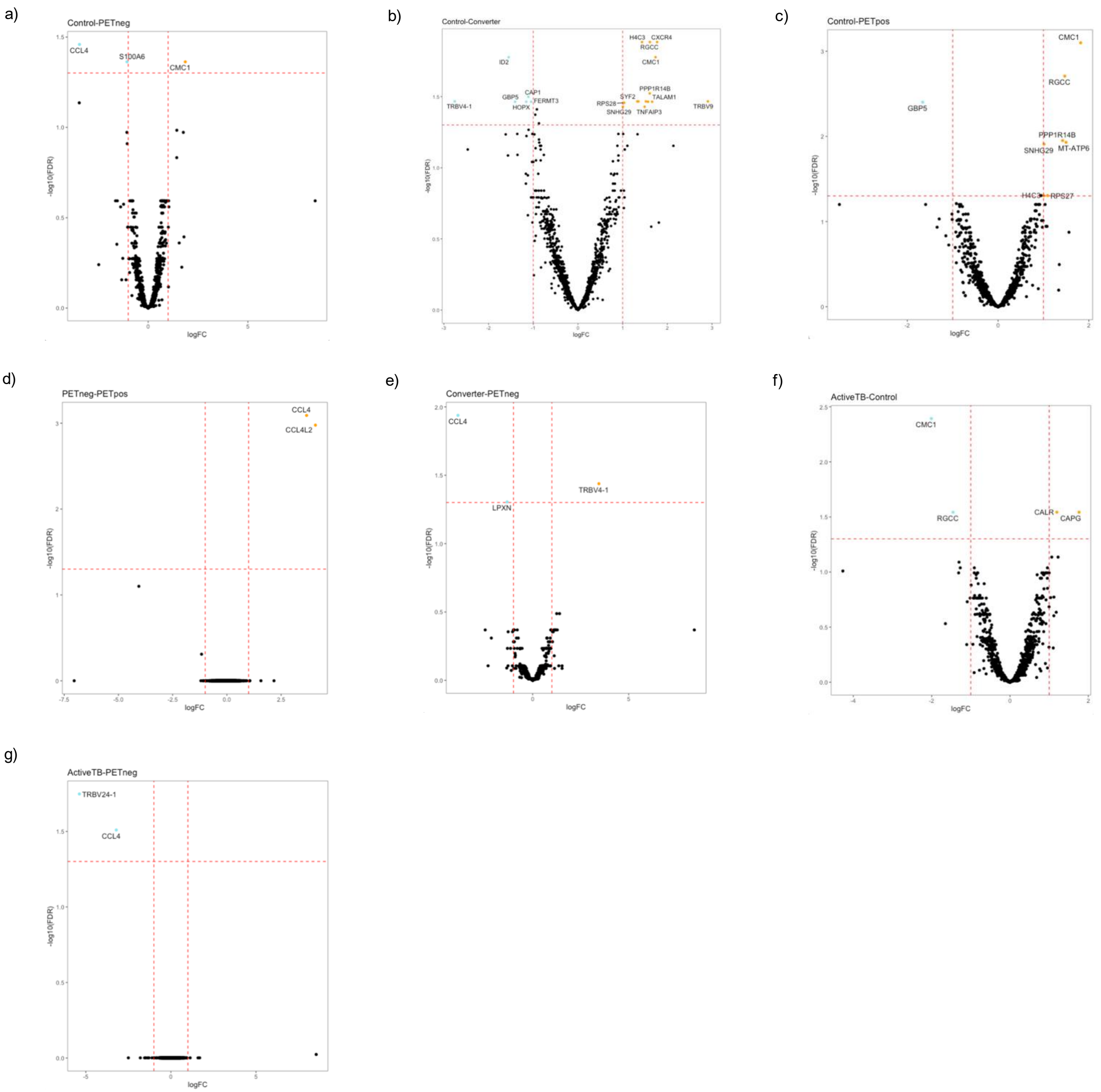

a)
Control-PETneg
CCL4
S100A6
CMC1
-log10(FDR)
logFC
b)
Control-Converter
H4C3
CXCR4
RGCC
CMC1
ID2
PPP1R14B
CAP1
GBP5
FERMT3
SYF2
TALAM1
TRBV4-1
HOPX
RPS28
SNHG29
TNFAIP3
TRBV9
-log10(FDR)
logFC
c)
Control-PETpos
CMC1
RGCC
GBP5
PPP1R14B
SNHG29
MT-ATP6
H4C3
RPS27
-log10(FDR)
logFC
d)
PETneg-PETpos
CCL4
CCL4L2
-log10(FDR)
logFC
e)
Converter-PETneg
CCL4
TRBV4-1
LPXN
-log10(FDR)
logFC
f)
ActiveTB-Control
CMC1
RGCC
CALR
CAPG
-log10(FDR)
logFC
g)
ActiveTB-PETneg
TRBV24-1
CCL4
-log10(FDR)
logFC

a)

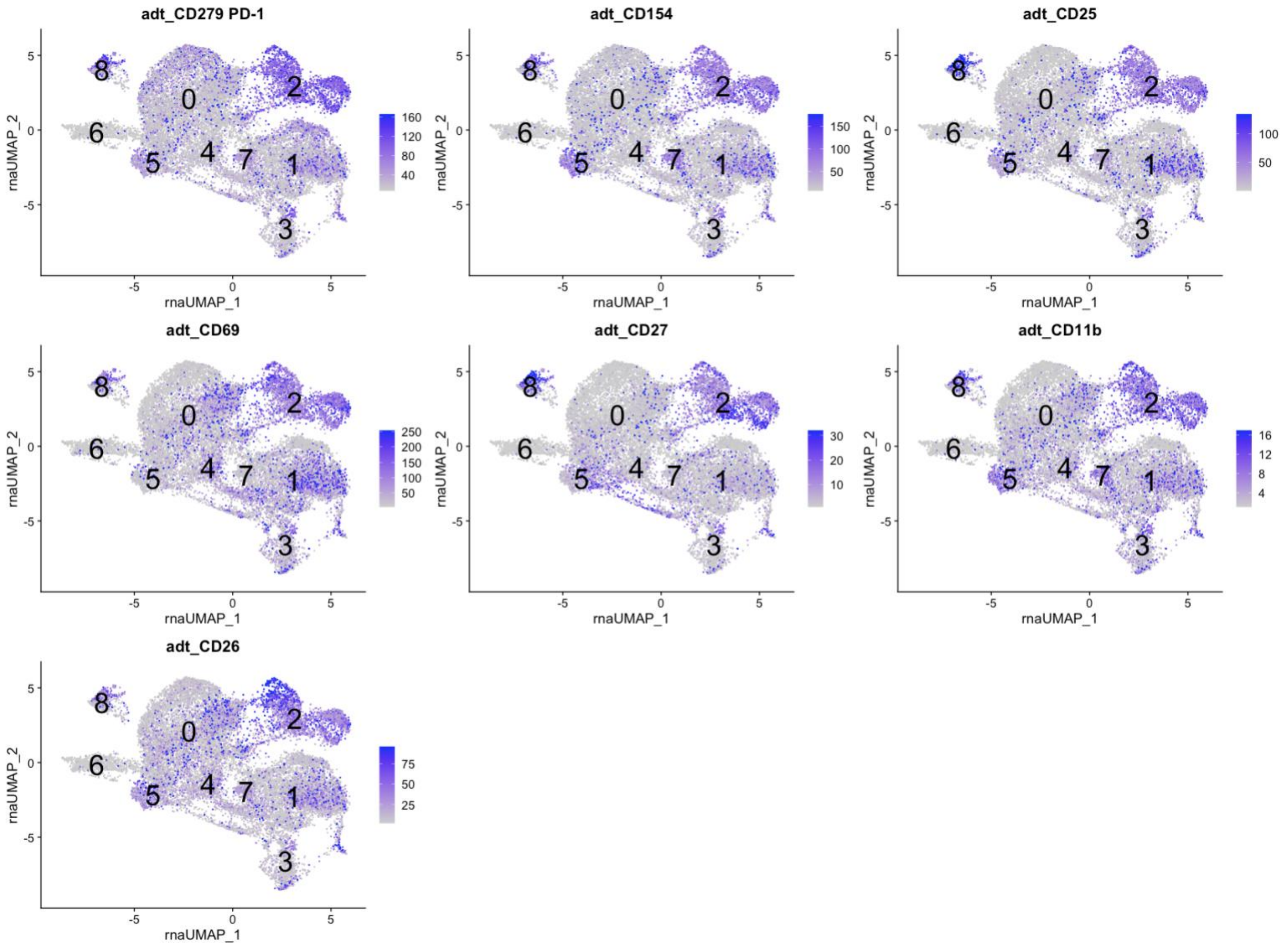
adt_CD279 PD-1
adt_CD154
adt_CD25
adt_CD69
adt_CD27
adt_CD11b
adt_CD26
rnaUMAP_1
rnaUMAP_2
160 120 80 40
150 100 50
100 50
250 200 150 100 50
30 20 10
16 12 8 4
75 50 25
0 1 2 3 4 5 6 7 8

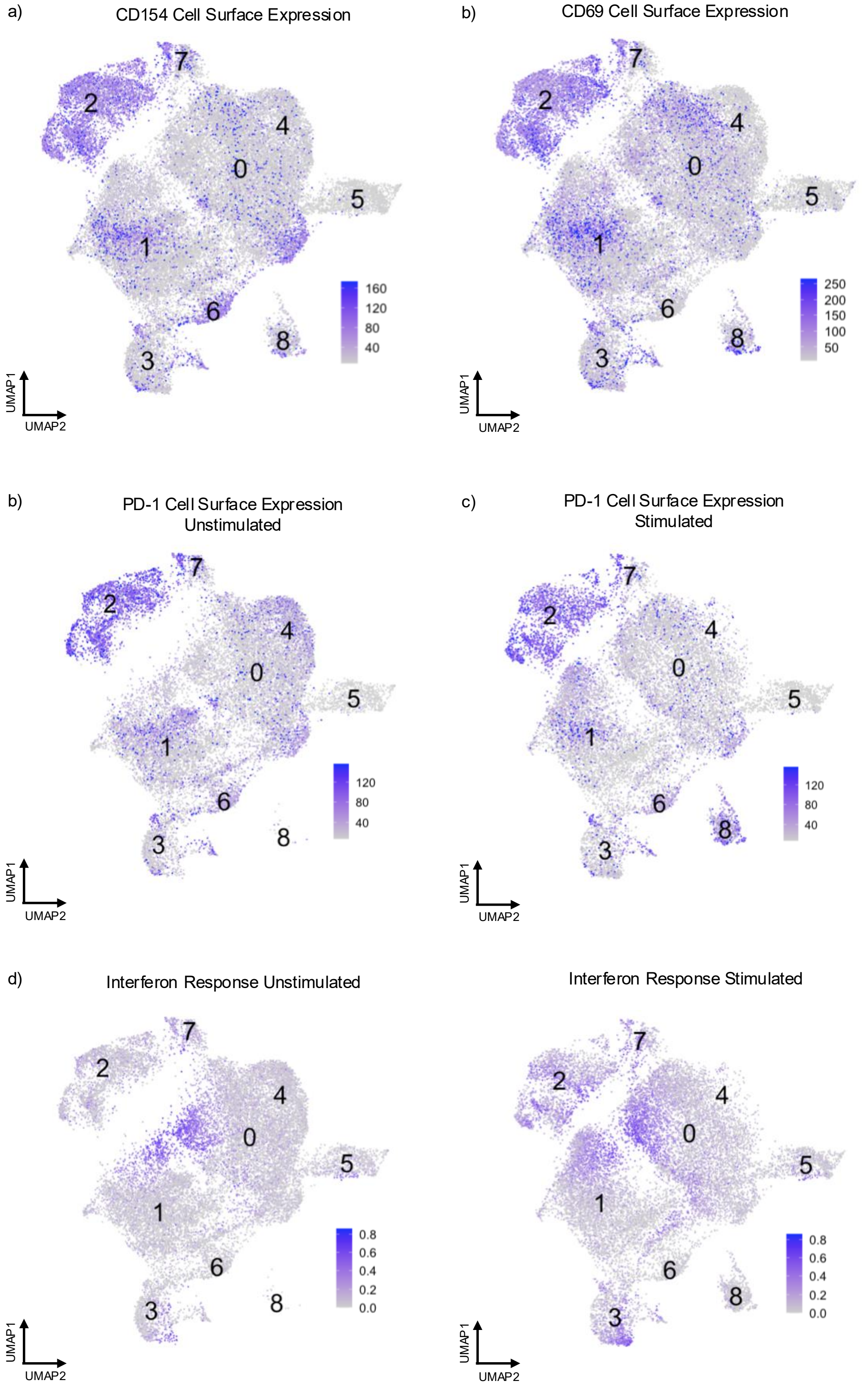

a)
CD154 Cell Surface Expression
b)
CD69 Cell Surface Expression
b)
PD-1 Cell Surface Expression
Unstimulated
c)
PD-1 Cell Surface Expression
Stimulated
d)
Interferon Response Unstimulated
Interferon Response Stimulated
0
1
2
3
4
5
6
7
8
160
120
80
40
250
200
150
100
50
0.8
0.6
0.4
0.2
0.0
UMAP1
UMAP2
